# Effective Early Treatment of *Microcystis* Exponential Growth and Microcystin Production with Hydrogen Peroxide and Hydroxyapatite

**DOI:** 10.3390/toxins15010003

**Published:** 2022-12-20

**Authors:** Ian Struewing, Nathan Sienkiewicz, Chiqian Zhang, Nicholas Dugan, Jingrang Lu

**Affiliations:** 1 Office of Research and Development, United States Environmental Protection Agency, Cincinnati, OH 45268, USA; 2 Department of Civil and Environmental Engineering, Southern University and A&M College, Baton Rouge, LA 70813, USA

**Keywords:** adsorption, H_2_O_2_, harmful cyanobacterial blooms, cyanotoxins, surface water, *Microcystis aeruginosa*, algicide, hydroxyapatite

## Abstract

Mitigating cyanotoxin production is essential to protecting aquatic ecosystems and public health. However, current harmful cyanobacterial bloom (HCB) control strategies have significant shortcomings. Because predicting HCBs is difficult, current HCB control strategies are employed when heavy HCBs have already occurred. Our pilot study developed an effective HCB prediction approach that is employed before exponential cyanobacterial growth and massive cyanotoxin production can occur. We used a quantitative polymerase chain reaction (qPCR) assay targeting the toxin-encoding gene *mcyA* to signal the timing of treatment. When control measures were applied at an early growth stage or one week before the exponential growth of *Microcystis aeruginosa* (predicted by qPCR signals), both hydrogen peroxide (H_2_O_2_) and the adsorbent hydroxyapatite (HAP) effectively stopped *M. aeruginosa* growth and microcystin (MC) production. Treatment with either H_2_O_2_ (10 mg·L^−1^) or HAP (40 µm particles at 2.5 g·L^−1^) significantly reduced both *mcyA* gene copies and MC levels compared with the control in a dose-dependent manner. While both treatments reduced MC levels similarly, HAP showed a greater ability to reduce *mcyA* gene abundance. Under laboratory culture conditions, H_2_O_2_ and HAP also prevented MC production when applied at the early stages of the bloom when *mcyA* gene abundance was below 10^5^ copies·mL^−1^.

## 1. Introduction

Harmful cyanobacterial blooms (HCBs) are the excessive growth of cyanobacteria in inland freshwater bodies during warm seasons. HCBs deplete dissolved oxygen and release a significant amount of cyanotoxins, disturbing aquatic ecosystems and causing illnesses and death to wildlife, livestock, and humans [1,2,3,4]. Cyanotoxins have toxic effects to the human liver, nervous, and gastrointestinal systems. These effects can be chronic or acute, and exposure can be through drinking water, surface water, or food consumption [5,6,7]. Cyanotoxins are also harmful to all levels of aquatic species, from aquatic invertebrates to shellfish and fish [8]. These cyanotoxins are resistant to drinking water treatment and can contaminate drinking water, threatening the health of drinking-water consumers. For example, in 2014, an HCB incident led to a water advisory and water supply shutdown in Toledo, Ohio, USA [9]. More alarming are the reports of HCBs occurring globally [10], with *Microcystis* blooms especially increasing temporally and geographically [11], as a consequence of increasing water temperatures and anthropogenic nutrient loading [12]. Altogether, HCBs pose serious public health, ecological, and economic risks. Therefore, effective HCB prevention strategies are essential to ensuring natural surface water quality and protecting public health and the environment. Developing effective, cost-efficient, and environmentally friendly control strategies will greatly benefit freshwater ecosystems and society [13,14,15,16].

Current HCB control strategies include treatment with chemical algicides, adsorption and flocculation, water column mixing, dredging, skimming, ultrasonication, and biotic treatments and controls [17]. Of these treatments, the most commonly deployed measures are the algicide hydrogen peroxide (H_2_O_2_) and adsorbents. Previous studies have shown that both treatments, H_2_O_2_ and adsorption via flocculation and sedimentation, can reduce cyanobacterial growth and toxin levels [18,19,20,21,22,23]. The caveat for those reported treatments is that they are often applied after HCBs are visible. Such treatments with algicides such as H_2_O_2_ during HCBs can lyse cyanobacterial cells and increase dissolved cyanotoxin levels in water [24,25]. Flocculation with alum and lime or other clay sediments has been used successfully with minimal negative environmental impact in various freshwater bodies [20,21,22,23]. However, the long-term treatment of lake HCBs with alum can also increase dissolved microcystin (MC) concentrations [26]. The main issue in those previous studies is that the measures were applied after HCBs were visible and negative impacts from the HCBs had already occurred. While these control strategies are relatively effective in various waterbodies, the key to successful HCB control is early detection and treatment, as mitigation efforts are more effective at the early onset of the blooms when cyanobacteria concentrations are still low [27]. Early control efforts also use lower and less harmful treatment concentrations, leading to less harmful side effects such as increased MC levels because of cell lysis and side effects of the treatment on other organisms. For instance, concentrations of H_2_O_2_ above 10 mg·L^−1^ can negatively impact other phytoplankton and zooplankton [19,28]. The negative impacts of the sedimentation of adsorbents (0.01 to 10 g·L^−1^) have been studied on shellfish and filter-feeding invertebrates [29], but it has been shown that very high concentrations (>1 g·L^−1^) are needed to negatively impact fish [30]. Nevertheless, H_2_O_2−_, adsorption-, and sedimentation-based approaches are the preferred treatments for *Microcystis* because they have been successfully used in the field and are environmentally friendly, with the by-products of H_2_O_2_ being oxygen and water. Cyanobacteria are also more sensitive to H_2_O_2_ than other phytoplankton [31]. Hydroxyapatite (HAP), Ca_10_(PO_4_)_6_(OH)_2_, which is naturally occurring in bones and shells and is known for its adsorptive properties [32], has been used to remove heavy metals and organic pollutants from water and soil [33]. HAP also adsorbs and removes bacteria as well as *Microcystis* from cultures [34,35], although cyanobacteria may also use HAP as a source of calcium and phosphate [36].

Early treatment of HCBs is the preferred option as it reduces or limits harmful side effects to other organisms; however, the key to effectively treating HCBs early is through the prediction or early warning of HCBs. Accurately predicting HCBs in freshwater bodies is a significant challenge. Tools to assess cyanobacterial activity and biomass range from microscopy, pigment extraction, quantitative polymerase chain reaction (qPCR), and measurements of biotic and abiotic factors, to satellite-based remote sensing [37]. Targeting the MC-producing gene *mcyA* using qPCR and reverse transcription quantitative polymerase chain reaction (RT-qPCR) provides toxin specificity as the technique targets a toxin-encoding gene (*mcyA*). Moreover, qPCR assays are sensitive and can detect low cell concentrations [38]. Previously, we demonstrated the concept of an early warning for HCBs [39]. This early warning system developed for HCB detection monitors the activities of multiple cyanotoxin-encoding genes of cyanobacteria (mainly *Microcystis*) using qPCR and RT-qPCR. This early warning system allows us to take timely HCB mitigation actions prior to the heavy growth of HCBs.

This study aimed at testing a proof of concept of an HCB control measure that can be deployed during early exponential cyanobacterial growth, as signaled by the microcystin-encoding gene (*mcyA*) qPCR. We hypothesized that both H_2_O_2_ and HAP could effectively control cyanobacterial overgrowth and MC production when applied at an early bloom stage signaled by qPCR and RT-qPCR. Considering that this proof of concept is based on our previous field studies [38,39], we needed to verify whether our previous key findings from the field studies could also be observed in and be consistent in laboratory-mimicked bloom events. We sought to confirm the following findings in the laboratory: (1) the presence of initial signals of *mcy* transcripts and abundance could be detected several days before MC detection, (2) *mcy* signals and total MC concentrations would have a significant correlation, and (3) control measures applied after the detection of *mcy* signals but prior to the onset of accelerating cyanotoxin production would significantly mitigate cyanobacterial and cyanotoxin accumulation.

## 2. Results and Discussion

### 2.1. Growth Curve of M. aeruginosa and Relationship between mcyA and MC

*M. aeruginosa* cultures were started with an initial cell concentration of either 10^3^ (high) or 10^2^ (low) cells·mL^−1^. To examine whether qPCR-determined *mcyA* gene abundance was correlated with the number of cells, microscopic cell counts were compared to gene abundance measured by qPCR. The log_10_-transformed microscopic cell count·mL^−1^ and log_10_-transformed qPCR-determined gene abundance had similar means (*p* = 0.094, paired *t*-test) and a strong linear correlation (*r* = 0.99, *p* = 0.004) (Figure S1). Previous studies also found the same strong linear correlation between *mcy* gene abundance and cell numbers [40,41], with each *Microcystis* cell containing only one copy of *mcyA* [42]. Thus, when discussing the number of cells in the culture, the growth rate, or the amount of MC produced per cell, we used hereinafter the gene copy number obtained from the *mcyA* qPCR assay.

**Figure 1:**
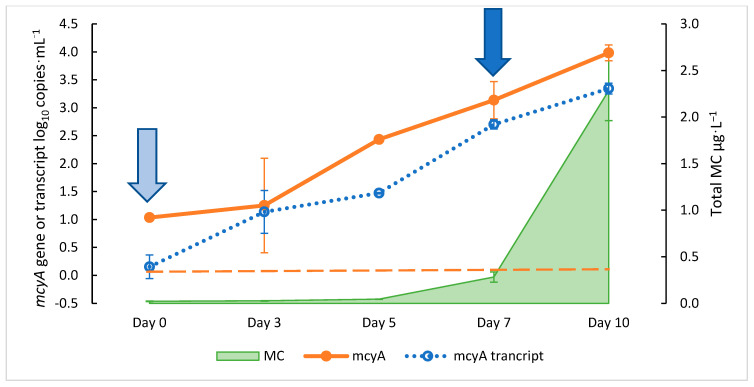
*mcyA* gene and gene transcript abundance versus microcystin (MC) concentration during the growth of *M. aeruginosa* culture. The arrow on the left indicates the beginning of *mcyA* gene signaling. The right arrow indicates the detection of the total MC production and time of the proposed treatment. Error bars indicate standard deviations of duplicate cultures. The graph indicates proliferation and expression of toxin producing gene seven days prior to MC concentrations exceeding the detection limit. The total MC lower limit of quantification (LLOQ) is 0.05 µg·L^−1^. The dashed orange line represents *mcyA* Limit of Detection (LOD) at 0.08 log_10_ copies·mL^−1^.

Rapid growth of *M. aeruginosa* occurred during a growth curve experiment mimicking a *M. aeruginosa* bloom with an initial concentration of 10^2^ cells·mL^−1^ and growing to a concentration of 10^5^ cells·mL^−1^ over 14 days. At the beginning of the culture (day zero), early signals of 10 copies·mL^−1^ *mcyA* gene and 2 copies·mL^−1^ of *mcyA* transcript were detected by qPCR and RT-qPCR, respectively (Figure 1). Both *mcyA* gene abundance and transcripts rapidly increased from day zero to day seven, increasing from 10^1^ to 10^3^ copies·mL^−1^ and from 2 to 5 × 10^2^ copies·mL^−1^ for gene abundance and the transcripts, respectively. The total MC was first detected above the lower limit of quantification (LLOQ) of the assay at 0.28 µg·L^−1^ on day seven (Figure 1). The *mcyA* gene abundance signals detected on day seven prior to the total MC detection were consistent with our previous findings during a natural HCB event, with the average *mcy* abundance being 3.20 and 3.70 log_10_ copies·mL^−1^, respectively, when the MC concentrations reached 0.3 µg·L^−1^ [38,39]. Additionally, the detection of both the *mcyA* gene and transcript signals seven days prior to MC detection confirmed the previous finding that *mcy* gene signaling is as an early warning of MC production. Thus, the growth curve confirmed three previous findings from the field studies: (1) the initial signals of *mcy* abundance and transcripts were detected before the total MC detection, (2) there was about a one-week lag time between the gene/transcript and total MC detections, and (3) the early exponential growth of *mcy* signals occurred one week after their initial detection.

**Figure 2:**
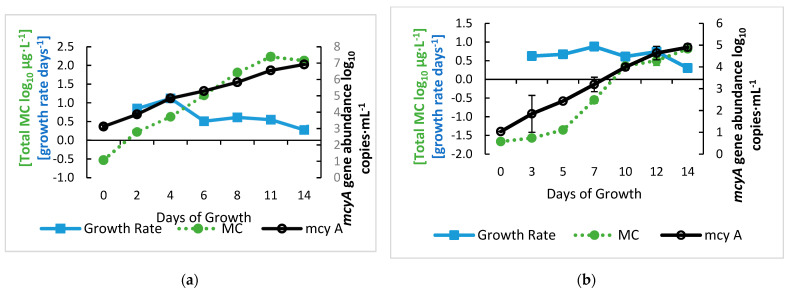
Growth curves of *M. aeruginosa* cultures showing the growth of *mcyA* gene abundance, total MC concentrations, and the growth rate of *Microcystis* cultures at (**a**) a high inoculation (10^3^ cells·mL^−1^) and (**b**) low inoculation (10^2^ cells·mL^−1^). Error bars represent standard deviations from duplicate samples.

Next, we determined at which point the increases in total MC began, as well as the general relationships between the total MC concentrations and *mcyA* abundance and transcripts. First, we examined the variation in growth rate to define the growth phases. The high- and low-inoculation cultures had similar specific growth rates with a mean of 0.65 ± 0.29 day^−1^ for the high-inoculation cultures and 0.64 ± 0.19 day^−1^ for the low-inoculation cultures (Figure 2). The high-inoculation cultures had a specific growth rate of 0.85 day^−1^ on day two and 1.12 day^−1^ on day four before dropping to the mean growth rate, while the low-inoculation cultures’ growth rates peaked on day seven with a specific growth rate of 0.88 day^−1^. The highest growth rates from our cultures occurred when the *mcyA* abundance was below 3 log_10_ copies·mL^−1^, which is consistent with previous reports that higher specific growth rates corresponded to lower cell densities [43,44,45,46]. Specific growth rates of *Microcystis* have been reported to be between 0.1 and 1 day^−1^, which can also vary among species and are influenced by light, temperature, and nutrient levels [43,44,45]. Growth rates of cyanobacteria in or isolated from natural surface waters tend to be slightly lower than those of pure laboratory cultures [45,46]. The average specific growth rate of *Microcystis* in Taihu Lake, China, was 0.42 day^−1^, and the in-situ growth rates ranged from 0.1 to 0.8 day^−1^ [46]. Both *mcyA* gene abundance and MC concentration had steady logarithmic growth, although the MC production lagged by five to seven days in the low inoculation growth curve (Figure 2b), suggesting that the significant elevation of MC was after the specific growth rates peaked (i.e., four days for low inoculation and seven days for high inoculation). By day 14, the specific growth rates of all cultures dropped to approximately 0.3 day⁻^1^, indicating that the cultures were in the late exponential growth phase by the end of the experiment. This growth curve indicated that the early logarithmic growth phase started after inoculating with a low cell density and experienced an initial lag phase for approximately three days before logarithmic growth, which then lasted for two weeks.

**Figure 3:**
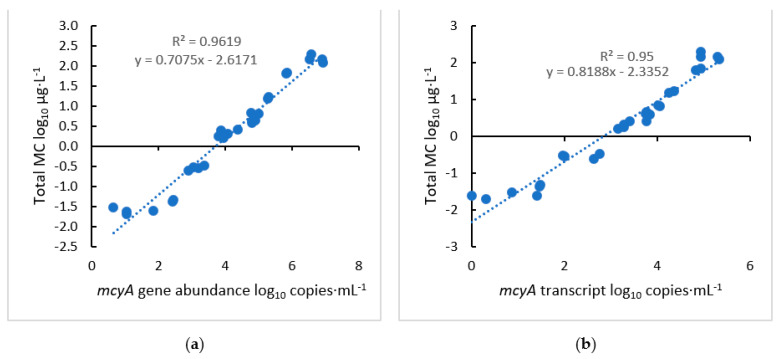
Regression of total MC concentration versus: (**a**) *mcyA* gene abundance and (**b**) *mcyA* transcript abundance.

The correlation analysis showed important findings that agreed with the previous field results [39]. First, significant positive correlations existed between log_10_-transformed MC concentrations and log_10_-transformed *mcyA* abundance (*r* = 0.98, *p <* 0.01) and transcripts (*r* = 0.97, *p <* 0.01) (Figure 3). Comparing MC versus log_10_-transformed *mcyA* abundance (Figure S2A) and transcripts (Figure S2B) indicated that an exponential increase in MC production, from a concentration of 6 μg·L^−1^ to greater than 120 μg·L^−1^, occurred when gene abundance was greater than 10^5^ copies·mL^−1^ or transcript abundance was greater than 10^4^ copies·mL^−1^. These findings further highlighted the relationship between MC and *mcyA* gene abundance, which also supports previous studies [47,48,49]. Other studies highlighted the complexity of the relationship between MC and the *mcyA* transcripts. One study showed that *mcyA* transcripts increased 70 times, while MC concentrations from culture increased 22 times, indicating a positive correlation [47]. On the other hand, a field study showed no correlation between the two [48]. Ngwa et al. [48] measured the relative expression of *mcyA* and noted that the transcription levels varied throughout the growth period, while the MC concentration increased constantly. It is possible that a similar correlation as we observed would have occurred if the total transcript levels of *mcyA* rather than the relative transcript levels were used. These *mcyA* abundance and gene expression results emphasize the importance of using *mcyA* as an “early” signal to initiate the treatment of MC production. For example, our finding that the exponential increase in MC production occurred when gene abundance reached a threshold of 10^5^ copies·mL⁻^1^, and this threshold was considered as the signal to employ control measures. Thus, the growth stage of greater than 10^5^ copies·mL^−1^ was considered to be MC-producing growth in this study (commonly considered as “overgrowth”). The use of the correlation between *mcyA* and MC to predict when control measures should be implemented is addressed below.

This growth curve experiment supported our previous findings from field studies that the *mcyA* gene is an early signal of MC production and that *mcyA* and MC are significantly correlated [38,39]. For instance, using the regression of log_10_ MC µg·L^−1^ versus the log_10_ *mcyA* copies·mL^−1^ obtained from the growth curve (Figure 3), the MC levels of 0.3, 1.6, and 8 µg·L^−1^ [50] corresponded to 2.96, 3.99, and 4.97 log_10_ copies·mL^−1^, respectively, which are in approximate agreement with the relationships obtained from the field samples (3.11, 4.20, and 5.22 log_10_ copies·mL^−1^) [39]. However, the 5.22 log_10_ copies·mL^−1^ reported by Lu et al. [39] corresponded to the 4 µg·L^−1^ MC level recommended by the World Health Organization (WHO) rather than the 8 µg·L^−1^ level by the U.S. Environmental Protection Agency (EPA) [50]. The U.S. EPA guidance levels for MC mentioned above are for the concentrations of MCs in drinking water at 0.3 µg·L^−1^ for bottle-fed infants and pre-school children, 1.6 µg·L^−1^ for school-age children and adults [50,51], and 8 µg·L^−1^ (4 µg·L^−1^, WHO) for MC exposure in recreational waters. The growth curves also showed the detection of *mcyA* gene abundance and signaling one week before the significant detection of MC (Figure 1), similar to results from our field studies [38,39]. Here, our emphasis is on the finding that our previous field results agree with those obtained in laboratory cultures, and we hypothesized that the initial detection and correlation of *mcyA* with MC can be used to signal the application of treatment measures to mitigate HCBs. The objective of this study was to build upon the basis of the timing concept of taking the early warning measurements, the onset of *mcyA* signaling, and the timing of preventative treatment. Thus, this study proposed a strategy to determine the timing of treatment. For drinking water and recreational water, the total MC advisory levels of 0.3 µg·L^−1^ and 8 µg·L^−1^ may be considered, respectively, and the timing of treatment can be set within an approximate week after the initial *mcyA* qPCR and RT-qPCR signals are detected. For drinking water, the advisory warning levels of *mcyA* qPCR and RT-qPCR signals are 10^3^ and 10^1^ copies·mL^−1^, respectively, and 10^5^ and 10^3^ copies·mL^−1^ for recreational water, respectively. The initial thresholds of qPCR and RT-qPCR signals suggested here were cited from a previous study in Harsha Lake, OH [39]. One week after the early warning signals were detected by qPCR and RT-qPCR, the cultures were treated with either H_2_O_2_ or HAP, testing the hypothesis that an early warning signal could be used as an indicator to begin treatment and prevent increases in MC concentrations and the exceedance of advisory thresholds. These threshold signals can change with advisory values such as state advisories or WHO guidance values and can be adapted to other lakes using the correlation equation of *mcy* genes with the total MC in a specific waterbody. The seven-day timing used in this study was just one selected time for the experiments by considering the above results and the practicality of weekly sampling frequency rather than it being the optimal time. For future field experiments, various time ranges should be tested on the treatment efficacy.

### 2.2. Treatment of Microcystis and MC with Hydrogen Peroxide

**Figure 4:**
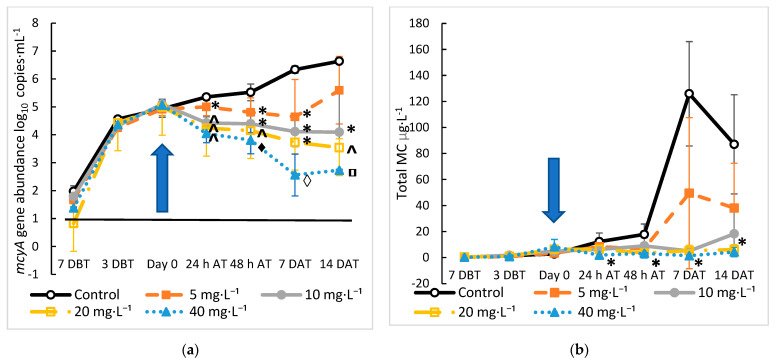
Effect of hydrogen peroxide treatment on: (**a**) *mcyA* gene abundance and (**b**) total MC concentration in *Microcystis* cultures grown for 21 days. Cultures were started seven days before treatment (DBT), and H_2_O_2_ was added on day zero, indicated by the arrow. For *mcyA*, * indicates the difference from the control; ^ indicates the difference from the control and 5 mg·L^−1^ dose; ♦ indicates difference from control, and 5 and 10 mg·L^−1^ doses; ◊ indicates difference from control, and 5, 10, and 20 mg·L^−1^ doses; and ⸋ indicates difference from control, and 5 and 20 mg·L^−1^ doses. For MC, * indicates significantly lower than the control for the 40 mg·L^−1^ treatment only 24 h after treatment (AT); 5, 20, and 40 mg·L^−1^ doses at 48 h AT; 10, 20, and 40 mg·L^−1^ doses 7 days after treatment (DAT) and 14 DAT. Error bars represent standard deviations of four samples. The solid black line indicates LOD for *mcyA* at 1.08 log_10_ copy·mL^−1^. The LLOQ for the total MC is 0.15 µg·L^−1^.

*M. aeruginosa* cultures were started with an initial *mcyA* abundance of 1.5 log_10_ copies·mL^−1^ and allowed to grow in LowN BG-11 for seven days prior to being treated with 5, 10, 20, or 40 mg·L^−1^ of H_2_O_2_. On the day of treatment, the average *mcyA* abundance of the cultures was 5 log_10_ copies·mL⁻^1^. An immediate decrease in cell density occurred at 24 h and 48 h, and cell numbers continued to decrease until 7 days after treatment (DAT) with all of the treatments being significantly lower than the control at 24 h, 48 h, and 7 DAT (*p* < 0.04) (Figure 4a). A dose-dependent effect on *mcyA* abundance, expression, and total MC was seen with the H_2_O_2_ treatments. Significant dose-dependent decreases from 5.06 to 4.64, 4.11, 3.72, and 2.56 log_10_ copies·mL^−1^ occurred 7 DAT for the 5, 10, 20, and 40 mg·L^−1^ treatments, respectively (Figure 4a). By 14 DAT, regrowth of *M. aeruginosa* was seen in cultures treated with 5 and 10 mg·L^−1^ H_2_O_2_, but not with higher doses. The *mcyA* abundance in the cultures treated with higher concentrations of H_2_O_2_ decreased continuously after treatment. These results confirmed previous studies on the treatment of *Microcystis* with H_2_O_2_. Laboratory experiments have shown a decrease in *Microcystis* concentration, as measured by photosynthetic pigments and oxidation activity 2 h after treatment with 5.1 and 10.2 mg·L^−1^ H_2_O_2_ [24], and a loss of cell integrity occurred after 3 h at H_2_O_2_ concentrations of 10 to 60 mg·L^−1^ [52]. Therefore, the immediate and significant decrease we detected at 24 h was expected. Yang et al. [19] also showed that *Microcystis* needed to be dosed with H_2_O_2_ concentrations above 2.7 mg·L^−1^ in the laboratory and 6.7 mg·L^−1^ in the field to prevent the regrowth of *Microcystis* five to seven days after treatment. The regrowth of *Microcystis* occurred more quickly in these experiments compared with our results, where the growth was suppressed for 14 days, but the starting cell concentrations were likely higher as the lab and field experiments were started with a *Microcystis* biovolume of > 10^7^ µm^3^·mL^−1^ [19]. The regrowth of cyanobacteria at doses lower than 5 mg·L^−1^ of H_2_O_2_ has also been reported by others [53,54]. H_2_O_2_ treatment of 0.1 mg·µg^−1^ chlorophyl *a* in a wastewater pond was more successful at reducing both cyanobacterial cell densities and MC concentrations by 60% to 70% (10^6^ cells·mL^−1^ and 1.4 µg·L^−1^ initial concentrations) for two weeks before regrowth occurred after three weeks and surpassed the initial concentrations by week four [54]. Our results indicate that *Microcystis* may need to be treated with concentrations higher than 5 mg·L^−1^ as 10 mg·L^−1^ was needed to prevent regrowth, which is more comparable to the concentration needed in environmental or field samples rather than pure cultures [19,53,55]. It would be tempting to use higher doses of H_2_O_2_ to control *Microcystis,* but higher doses can negatively affect other plankton. For instance, H_2_O_2_ treatments at a concentration of 20 mg·L^−1^ negatively affect zooplankton [19], and treatments above 10 mg·L^−1^ decrease diatom and green algae abundance [28]. It has also been shown in mesocosm studies that treatment at 10 mg·L^−1^ can reduce cyanobacterial growth, increase phytoplankton abundance and diversity, and prevent cyanobacterial regrowth [28,55,56]. Therefore, our results combined with previous studies suggest an appropriate treatment level of H_2_O_2_ on *Microcystis* dominated HCBs should be between 5 to 10 mg·L^−1^.

**Figure 5:**
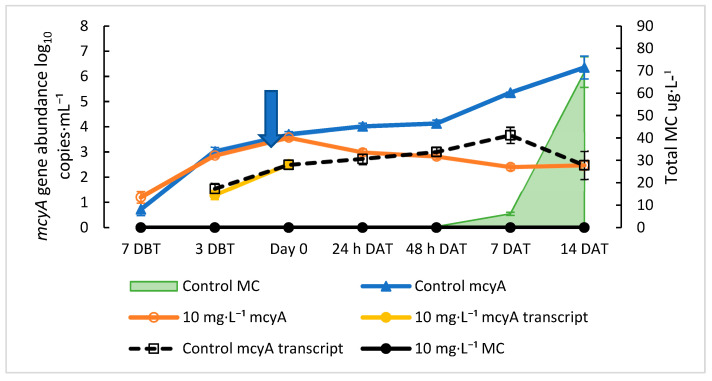
Effect of 10 mg·L^−1^ hydrogen peroxide treatment on *M. aeruginosa* applied at low cell concentrations (3.57 log_10_ copies·mL^−1^) after the onset of *mcyA* gene signaling. Cultures were started at seven DBT. The arrow represents the start of treatment on day zero. Error bars represent standard deviations of triplicate samples. Treatment resulted in the immediate decrease in *mcyA* gene abundance, and *mcyA* transcript levels in the treatment cultures dropped below detection (no transcripts detected between day zero and 14 DAT). The concentrations of total MC in the treated cultures were undetectable at day zero (below 0.15 µg·L^−1^) before treatment and remained undetectable after treatment, whereas the control MC concentrations increased to 70 μg·L^−1^. The LOD for *mcyA* is 0.08 log_10_ copies·mL^−1^, and the LLOQ for total MC is 0.15 µg·L^−1^.

The dose-dependent decreases in the total MC concentration in the treated cultures were also observed after the H_2_O_2_ treatments. Overall, the reduction in total MC concentration by H_2_O_2_ treatments followed the same trend as the reduction in *mcyA* abundance, although MC was reduced less than *mcyA* abundance. The decrease in total MC concentration 48 h after treatment ranged from 49% to 82% compared with an 80% to 98% reduction in *mcyA* abundance. These reductions in *mcyA* abundance and MC concentration are comparable to in-lake treatments with H_2_O_2_ that reduced the cyanobacterial concentration by 99% and 97% (6 × 10^5^ cells·mL^−1^ initial concentration), while MC concentrations (20 µg·L^−1^ initial concentration) were reduced by similar amounts or to an undetectable level [18,57]. At 48 h, our control cultures had a similar cell concentration of 2.2 × 10^5^ cells·mL^−1^ and MC concentration of 17.8 µg·L^−1^. These treatments were at a lower concentration of H_2_O_2_ (2 to 3 mg·L^−1^) than needed in our experiments to reduce *Microcystis* growth. However, these treatments were applied to *Planktothrix*, *Aphanizomenon*, and *Dolichospermum* dominated blooms [10,49], which have been shown to be more sensitive to H_2_O_2_ treatment than *Microcystis* [18]. However, a similar in-lake treatment of a *Planktothrix* bloom decreased cell concentrations by 80% (from 1780 to 358 filaments·mL^−1^), but a constantly low level of MC (0.4 to 1.5 ppb) was detected throughout the experiment [58]. This shows that treatments and results may have to be adjusted based on the characterization of the cyanobacterial population. Control cultures had total MC concentrations of 17.80 µg·L^−1^ 48 h after treatment, while the total MC concentration in treated cultures was 6.82, 8.95, 3.48, and 3.23 mg·L^−1^ for the 5, 10, 20, and 40 mg·L^−1^ H_2_O_2_ treatments, respectively (Figure 4b). The total MC concentrations showed a significant decrease for all of the treatment groups compared to the control 48 h after treatment (*p* < 0.05) (Figure 4b). The total MC levels never recovered to the level in the control cultures. However, the total MC concentrations in the 5 mg·L^−1^ of H_2_O_2_ cultures showed increasing signs on 7 and 14 DAT, becoming not significantly different from the control (*p* > 0.10) (Figure 4b). According to previous studies, the main mechanism by which H_2_O_2_ reduces the total MC levels is by inducing cell damage and death and reducing *Microcystis* cell growth [24,59]. It has also been shown that H_2_O_2_ is relatively weak at degrading MC, removing only 17% of MC-LR after a 60-min treatment [60]. An often-cited negative consequence of H_2_O_2_ treatments is the release of MC from damaged cells, increasing the MC concentration in water. Several studies have noted increases in extracellular MC concentration several days after H_2_O_2_ treatments in mesocosms and laboratory experiments [19,24,54,61]. Interestingly, MC concentrations increased significantly more in *Microcystis* colonies of the greatest size when treated with H_2_O_2_, showing the importance of early treatment before large colonies form [61]. Such a consequence was absent in the current study because the total MC was not massively produced. For instance, the total MC concentrations with an initial mean concentration of 4.99 ± 0.08 µg·L^−1^ did not increase after treatment with H_2_O_2_ until 7 DAT in the 5 mg·L^−1^ treatment and 14 DAT in the 10 mg·L^−1^ treatment, while the total MC concentrations did not increase in the higher H_2_O_2_ concentrations. These results show that H_2_O_2_ effectively prevented *Microcystis* growth and MC production if the treatment was conducted during an early exponential growth phase. Treatment in the early exponential growth phase before the total MC levels have increased is important, as the primary action of H_2_O_2_ treatment is preventing *Microcystis* growth and thus new MC production. To confirm these results, an optimized experiment was conducted with an initial *mcyA* abundance of 0.96 log_10_ copies·mL^−1^ of culture grown in LowN BG-11. The treatment with 10 mg·L^−1^ H_2_O_2_ was planned on day seven of culture because on day seven, the culture was in early exponential growth. At the beginning of the experiment, the *mcyA* signal was initially detected by qPCR (1.2 log_10_ copies·mL^−1^) and the *mcyA* transcript signal was detected by RT-qPCR (1.1 log_10_ copies·mL^−1^) four days after the inoculation (Figure 5). At the time of treatment (day zero) with *mcyA* abundance of 3.6 log_10_ copies·mL^−1^, the total MC was detectable at 0.13 ug·L^−1^ just above the limit of detection (LOD) of 0.10 μg·L^−1^ but below the LLOQ of 0.15 μg·L^−1^. The total MC became undetectable after treatment and remained so throughout the experiment (14 days), while the total MC concentration increased to 70 μg·L^−1^ in the control cultures. *mcyA* gene abundance decreased immediately upon treatment to 2.41 log_10_ copies·mL^−1^ by 7 DAT and 2.47 log_10_ copies·mL^−1^ by 14 DAT (Figure 5). Together, these results demonstrated that treating *M. aeruginosa* at its early exponential stage signaled with *mcyA* abundance and/or transcripts would prevent MC-producing growth and keep MC production from ultimately reaching even the lowest drinking water warning level of 0.3 μg·L^−1^ MC [51] and preventing regrowth for at least 14 days.

### 2.3. Mitigation of Microcystis and MC Using Hydroxyapatite

Considering adsorption is a successful measure to mitigate *Microcystis* and MC [22,23], HAP was chosen as an adsorbent surrogate for this study because of its high adsorption properties and availability. Preliminary experiments were conducted to test the adsorption of *M. aeruginosa* cells and total MC across different types and sizes (60 nm, 200 nm, and 40 µm) of HAP particles (Figures S3 and S4). On the basis of the initial experiments using various HAP particle sizes, subsequent treatment studies used 40 µm HAPs at concentrations of 0.5 g·L^−1^ and 2.5 g·L^−1^. Practically, the application of 40 µm HAPs can reduce the cost and inhalation risk compared with the nanoparticles (e.g., 60 nm and 200 nm).

**Figure 6:**
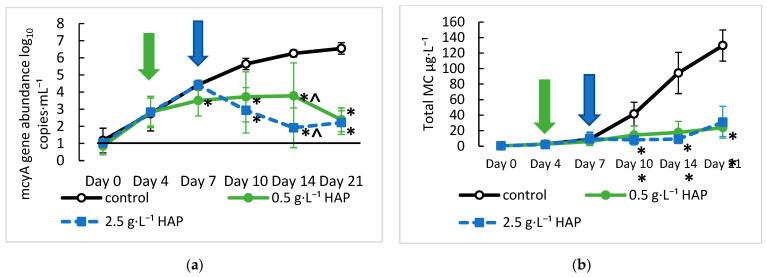
Effects of hydroxyapatite (HAP) treatment on: (**a**) *mcyA* gene abundance and (**b**) total MC concentration in *Microcystis* cultures grown for 21 days. Treatment began on day four for 0.5 g·L^−1^ HAP (left arrow) and day seven for 2.5 g·L^−1^ HAP (right arrow). * Indicates a significant difference from the control (*p* < 0.05), and ^ indicates a significant difference between treatments (*p* < 0.05). Error bars represent the standard deviations of six samples. The solid black line indicates LOD for *mcyA* at 1.08 log_10_ copy·mL^−1^. LLOQ for the total MC is 0.15 ug·L^−1^.

Cultures of *M. aeruginosa* were grown in LowN BG-11 at an initial concentration of 1.01 log_10_ copies·mL⁻^1^. They were treated with 40 µm HAP particles at either 2.5 g·L^−1^ on day seven (0.35 g total) or with 0.5 g·L^−1^ on days 4, 7, 10, and 14 (0.26 g total). The HAP particles showed significant adsorption of *M. aeruginosa*, and cultures treated with HAP showed decreasing cell and total MC concentrations. Both treatment levels of HAP removed more than 99% of detectable cells on day 14 compared with the control (i.e., no HAP addition) (Figure 6a). Likewise, on day 14, HAP reduced the total MC level in the cell cultures by 81.2% and 90.3% for the low (0.5 g·L^−1^) and high (2.5 g·L^−1^) HAP concentrations, respectively (Figure 6b). Treatment with HAP caused an immediate decrease in *mcyA* abundance. The treatment with 0.5 g·L^−1^ HAP on day four resulted in a 1-log_10_ reduction of *mcyA* by day seven compared with the control (*p* = 0.03), and treatment on day seven with 2.5 g·L^−1^ HAP resulted in a 2-log_10_ reduction in *mcyA* compared with the control by day 10 (*p* = 0.001) (Figure 6a). This immediate adsorption of *Microcystis* cells by HAP is consistent with previous results, in which the highest removal rates of cells treated with clays/minerals occurred within the first 10 min of treatment, which would be necessary for environmental treatment [62]. The adsorption of cells to the surface of HAP would bind and remove cells akin to the process of flocculation with clays and minerals that remove cyanobacteria as they settle to the bottom of the water column, resulting in cell mortality [63,64]. The concentration of *mcyA* in treated cultures remained significantly lower than the control throughout the treatment, with the concentration of *mcyA* being close to 2.3 log_10_ copies·mL^−1^ for both 0.5 g·L^−1^ and 2.5 g·L^−1^ HAP treatments on day 21 compared with 6.54 log_10_ copies·mL^−1^ for the control, effectively preventing *M. aeruginosa* regrowth. Treatment with a one-time dose of HAP at 2.5 g·L^−1^ showed a larger reduction in *mcyA* abundance on days 10 and 14 than the sequential dosing of HAP at 0.5 g·L^−1^. *mcyA* abundance was significantly lower (*p* = 0.034) on day 14 at 1.9 log_10_ copies·mL^−1^ for 2.5 g·L^−1^ and 3.78 log_10_ copies·mL^−1^ for 0.5 g·L^−1^ (Figure 6a). These results suggest that the reduction in *mcyA* abundance and thus cell adsorption corresponded to the total amount of HAP added to the cultures. On days 10 and 14, the total amount of HAP added to the 2.5 g·L^−1^ cultures was 0.35 g compared with 0.15 g and 0.21 g on days 10 and 14 for the 0.5 g·L^−1^ cultures, respectively. By day 21, the total amount of HAP added was 0.35 g for the 2.5 g·L^−1^ cultures and 0.26 g for the 0.5 g·L^−1^ cultures, and as the total amount of HAP increased in the sequentially dosed culture, it adsorbed more cells and reduced the amount of *mcyA* detected. By day 21 of the experiment, cultures treated with both doses of HAP had *mcyA* abundances of approximately 2.3 log_10_ copies·mL^−1^ and total MC concentrations of 24 μg·L^−1^ for 0.5 g·L^−1^ HAP and 31 μg·L^−1^ for 2.5 g·L^−1^ HAP.

The general effect of HAP treatment on the total MC levels of the cultures was similar to that of the adsorption and reduction of *M. aeruginosa* cells, but the total MC reduction was less compared with that of the cells. By day 10, the total MC levels of both HAP treatments were significantly lower than the control (*p* < 0.05) and remained relatively low throughout the experiment (Figure 6b). The treatments with the two HAP concentrations showed similar efficacies of MC mitigation. For example, the concentration of total MC was lower on days 10 and 14, but higher on day 21 in the 2.5 g·L^−1^ cultures than in the 0.5 g·L^−1^ cultures, but their efficacies showed no significant difference (*p >* 0.1). The major difference between the reduction in total MC and the reduction in *mcyA* abundance was the level of reduction. From days 10 to 21, HAP treatment reduced the total MC levels by 65% to 92% with the largest reduction in total MC in the 2.5 g·L^−1^ treatment on day 14, whereas the reduction in *mcyA* abundance ranged from 2 to 4-log_10_ units, or a greater than 99% reduction. These results suggest that the principal mechanism through which HAP reduces the total MC levels is through adsorbing *Microcystis* cells and removing them from the culture through sedimentation, reducing the growth as evidenced by the significant decrease in *mcyA* abundance compared with the decrease in total MC concentrations. Additionally, the results suggest that the reduction in MC levels does not correlate with the amount of HAP added to the cultures, as both the 0.5 g·L^−1^ and 2.5 g·L^−1^ cultures had comparable levels of total MC. Our results indicate that the adsorbent HAP has a greater affinity for the adsorption of *M. aeruginosa* cells than the adsorption of MC. The concentration of HAP in our experiments was in the range of those typically tested with clay adsorbents (0 to 5 g·L^−1^). Our results confirmed what others have shown, in that cyanobacterial removal increases with increased concentrations of the adsorbent but often does not significantly increase after a threshold of adsorbent (0.5 to 1 g·L^−1^) is reached [62,63,65]. Our removal efficacy of 99% of cells was slightly higher than what others have reported, but some types of clay removed up to 90% of cells at these adsorbent concentrations [62,63]. Our removal efficacy may have been higher because our experiments took place over an extended time period (14 days) as opposed to the removal of cells measured over hours [62,63]. In these tests, clay minerals could remove 10^6^ cells·mL^−1^ of *Microcystis* cultures at 0.75 g·L^−1^ [63], and 26 clay/minerals were tested with an initial *Microcystis* cell concentration of 10^9^ cells·mL^−1^, with removal efficacies that ranged from less than 50% to 90% at concentrations of 0.7 g·L^−1^ [62]. While the removal efficacy by HAP was higher, the total number of cells removed by these clay minerals could be greater and the efficacy of removal between different minerals could vary widely. In the natural environment, the cells would adsorb to HAP or other clay sediments, where they would settle into the bottom of the waterbody where both the cells and MC are degraded [66]. Our cultures may have retained higher MC levels because, although the cells were adsorbed to the HAP particles on the bottom of the flask, the cells still received light and nutrients in the small batch culture conditions and thus may have remained viable and released MC. To reduce the possibility of MC release from HAP sediments, as occurred in our culture experiment, it is necessary to treat the HAB event at a very early stage of MC production. We were aware that HAP sediments could release phosphorus into the water column and promote algal growth [36,67]. Thus, while we used HAP in this preliminary study, we have recognized that there are potential downsides related to phytoplankton growth. In future studies, other types of particles for adsorption/flocculation in real world scenarios should be assessed.

### 2.4. Comparison of Treatment with HAP versus Hydrogen Peroxide

**Figure 7:**
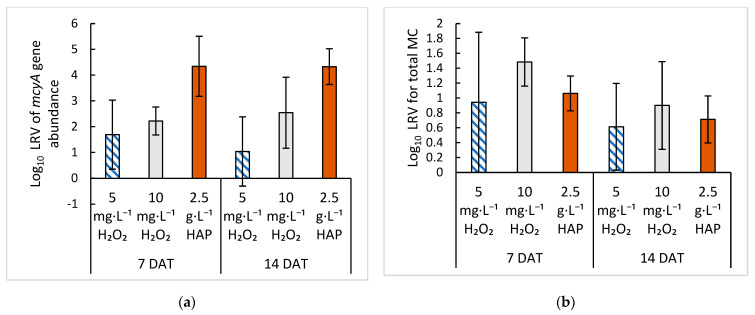
Comparison between the impacts of HAP and H_2_O_2_ treatment at 7 and 14 DAT using log_10_ reduction values (LRV). (**a**) Treatment effect on the LRV in *mcyA* gene abundance and (**b**) total MC concentration compared with the control cultures. Error bars represent standard deviation of replicate samples (*n* = 4 for H_2_O_2_ and *n* = 6 for HAP).

Treatments of *Microcystis* cultures with either HAP or H_2_O_2_ prevented MC-producing growth lowering *mcyA* abundance, *mcyA* transcript level, and MC concentration. It would be beneficial to know which treatment was more effective at preventing *Microcystis* growth and thus HCBs. To determine if there was a difference in the effectiveness of these two treatments, we compared the results from the 2.5 g·L^−1^ treatment of HAP and the 5 and 10 mg·L^−1^ treatments of H_2_O_2_ on 7 and 14 DAT. These were chosen because the 2.5 g·L^−1^ HAP and H_2_O_2_ treatments were applied on day seven of culture growth and the same measurements were taken on the cultures at both the 7 and 14 DAT time points. When comparing the number of cells in the cultures after treatment, HAP led to a 4-log_10_ reduction in *mcyA* abundance at 7 DAT, while the H_2_O_2_ treatments led to a 1.69-log_10_ reduction at 5 mg·L^−1^ and 2.22-log_10_ reduction at 10 mg·L^−1^ at 7 DAT. While all of the treatments showed a large reduction in *mcyA* abundance, treatment with HAP resulted in a lower number of *mcyA* gene copies in cultures at both 7 and 14 DAT compared with the H_2_O_2_ treatments (Figure 7a). Although HAP reduced the number of cells in the culture more than the H_2_O_2_ treatments, the same effect was not seen for MC concentrations, where 10 mg·L^−1^ H_2_O_2_ was the most effective at reducing MC concentrations (Figure 7b). Our results indicate that an adsorbent such as HAP may be more effective at removing cells from the culture, but H_2_O_2_ is more effective at preventing the total MC increase. It should be noted, however, that because of the nature of treatment in a culture flask, where HAP has longer and more constant contact with the cells compared with a treatment that takes place in a lake or reservoir, the removal of *Microcystis* cells could artificially increase in the culture experiments. Treatments of HCBs with H_2_O_2_ or flocculation with clay sediments in both mesocosms and the field have shown to be effective at reducing the cell numbers and MC concentrations. Matthijs et al. [18] found a 99% reduction in cyanobacterial concentration (6 × 10^5^ cells·mL^−1^ initial concentration) for an in-lake treatment with 2 mg·L^−1^ of H_2_O_2_. Yang et al. [19] reported a 90% reduction in MC concentrations (4 µg·L^−1^ initial concentration) with more than 6.7 mg·L^−1^ H_2_O_2_. These reduction levels found with H_2_O_2_ are significantly higher than those reported for flocculation and sedimentation, where reductions of MC concentrations ranged from 40% to 50% in a lake treated with 40 to 50 g·m^−2^ of modified local soil when compared with an untreated section of the lake [23]. Our results with HAP showed a larger reduction at 65% to 92%. However, the lake treatments were applied to a high cell biomass where a 1 cm thick HAB had already formed in the lake [23]. When modified clay was applied in Xuanwu Lake in Nanjing, China, at cell concentrations of 2.7 × 10^7^ cells·mL^−1^, the cell concentrations were reduced to 6 × 10^3^ cells·mL^−1^, and MC concentrations were reduced to < 0.01 µg·L^−1^ from 0.03 to 0.62 µg·L^−1^ [22], further showing the importance of early treatment. As the conditions of these studies varied, it is difficult to compare the results of these two types of treatments. Given that they have similar positive outcomes, it would be beneficial to study them simultaneously in a field environment to see if one is more effective than the other, or if they could be combined for more effective mitigation and prevention of HCBs.

## 3. Conclusions

To assess the optimal timing of treatment for an HCB event, this study examined the growth curve, growth rate, and relationships of MC production with *mcyA* gene abundance and expression during the logarithmic growth phase of *M. aeruginosa* in a lowN medium with low inoculation. The optimal timing of treatment was in the early logarithmic growth phase, which was characterized by high growth rates, pre-exponential MC production, and significant correlations between MC production and *mcyA* abundance. We found that the optimal timing for treatment was approximately seven days after inoculating the cultures because the cell growth was in the early logarithmic phase and MC concentrations did not exceed the U.S. EPA recommended recreational water limit (8 µg·L^−1^). To determine the optimal timing, *mcyA* qPCR signal was used as an indicator, and an abundance of less than 10^5^ copies·mL^−1^ signaled early-stage treatment to mitigate MC-producing growth and MC production. Treatment measures, including the oxidant H_2_O_2_ and the adsorbent HAP, taken at the early stage of growth, demonstrated effective mitigation and control of the exponential growth of *M. aeruginosa* and toxin production without regrowth at low treatment concentration levels. Our results provide evidence that early treatment of HCBs with hydrogen peroxide or HAP are promising approaches in mitigating or controlling toxin-producing cyanobacteria and their respective cyanotoxins. In addition, these two treatment approaches are cost-effective and can be feasible to manage (source or recreational) waterbodies and decrease ecosystem and public health risks associated with HCBs.

## 4. Materials and Methods

### 4.1. Microcystis aeruginosa Cultivation and Growth Curve Experiments

Stock cultures of *M. aeruginosa* were grown in BG-11 (Cyanobacteria BG-11 Freshwater Solution, MilliporeSigma, Burlington, MA, USA) to a cell density of approximately 10^7^ cell·mL^−1^ in 300 mL glass flasks. The cultures were grown in an environmental chamber with a light intensity of 44.02 µmol·m^−2^·s^−1^ from cool white fluorescent lights measured using a LICOR LI-1500 at the surface of the culture flasks with a 16/8-hour light/dark cycle and at 25 °C. Additionally, the cultures were provided with air from a laboratory air line for the growth curve experiments. The experiments described below used the same conditions as the stock cultures above. The growth curve cultures were inoculated with 10^3^ cells·mL^−1^ (high inoculation) or 10^2^ cells·mL^−1^ (low inoculation) from stock *M. aeruginosa* cultures. For cultures with low inoculation densities, a modified LowN BG-11 (LowN) medium, containing 1% of the sodium nitrate in BG11 but with all other ingredients being equal to standard BG-11 (Table S1), was used. Duplicate 1 L cultures were grown in 2 L glass flasks and sampled every two or three days. Of each collected sample, 1 mL was used for direct cell counting using a microscope and hemacytometer, 10 to 100 mL was filtered through 0.8 µm Isopore polycarbonate filters (Millipore-Sigma, Burlington, MA, USA) and stored in Lysing Matrix A bead tubes (MP Biomedicals, Irvine, CA, USA) with Trizol (Invitrogen, Waltham, MA, USA) at −80 °C for RNA/DNA extraction, and 10 mL was collected in a glass centrifuge tube and stored at −20 °C for the total MC enzyme-linked immunosorbent assay (ELISA) measurement. Two growth curve experiments with duplicate cultures were performed using LowN and were grown for 14 days. The sampling and sample processing mentioned here were also used for the treatment experiments described below.

### 4.2. H_2_O_2_ Treatment of M. aeruginosa

To test the effect of different concentrations of H_2_O_2_ on *Microcystis* growth and toxin production, 200 mL cultures were started at a concentration of approximately 10^4^ cells·mL^−1^ in LowN medium and grown for seven days prior to H_2_O_2_ treatment, at which time they were dosed with 5, 10, 20, or 40 mg·L^−1^ of H_2_O_2_. The H_2_O_2_ concentration was measured using absorbance at 240 nm with a Spectramax spectrophotometer (Molecular Devices, San Jose, CA, USA). The experiment was repeated with cultures grown in duplicate for each treatment. Samples were taken at 7, 3, and 0 days before treatment (DBT), and 24 h, 48 h, 7, and 14 days after treatment (DAT). As described above, the samples were taken for DNA/RNA extraction and total MC analysis by ELISA. Treatment of low inoculation cultures was done using 10 mg·L^−1^ H_2_O_2_. For the low inoculation test, triplicate 1 L cultures were grown in 2 L glass flasks with LowN medium with lab air. Samples, as described above, were taken for DNA/RNA and total MC analyses; however, 100 mL was filtered through 0.45 µm polycarbonate filters (PALL Corporation, Port Washington, NY, USA) for DNA/RNA extraction. A summary of the *M. aeruginosa* test conditions can be found in Table S2.

### 4.3. HAP Adsorption of M. aeruginosa and MC

A preliminary study was conducted to examine whether HAP particles were an effective adsorbent for *M. aeruginosa* and MC, and which HAP particle size had the best adsorption properties. HAP particle sizes at 60 nm (MK Nano Technologies, Japan), 200 nm (Sigma-Aldrich, St. Louis, MO, USA), and 40 µm (Bio-Rad Laboratories, Hercules, CA, USA) were used for these initial experiments. The experiment started with 200 mL cultures at an inoculation of 5 × 10^3^ cells·mL^−1^ of *M. aeruginosa* grown in 500 mL flasks for 21 days. Cultures were grown in LowN for seven days prior to HAP treatments at 0.5 g/L or 2.5 g/L. All of the treatments were initiated on day seven. Samples were taken on day 0, 4, 7, 10, 14, and 21. The experiment was repeated using 40 µm HAP particles at concentrations of 0.25, 0.5, and 2.5 g·L^−1^, as described above.

To test the adsorption of *M. aeruginosa* to HAP particles, 200 mL cultures were started in LowN at a concentration of 10^4^ cells·mL⁻^1^. Samples were taken on day 0, 4, 7, 10, 14, and 21, with 20 mL being removed each time for DNA/RNA extraction and total MC analysis, as described above. The remaining 140 mL cultures were then treated with 40 µm HAP particles at either 2.5 g·L^−1^ on day seven or sequentially dosed with 0.5 g·L^−1^ at day 4, 7, 10, and 14 with the total amount of HAP added being 0.35 g for the 2.5 g·L^−1^ concentration compared to 0.26 g for the 0.5 g·L^−1^ concentration. The experiment was repeated with triplicate culture samples for each treatment.

### 4.4. DNA/RNA Extraction

RNA/DNA was isolated from the filtered *M. aeruginosa* cells using TRIzol reagent (Life Technologies, Carlsbad, CA, USA) following the manufacturer’s protocol. Briefly, the samples were thawed and then bead beaten for 1 min using a Mini-Beadbeater-16 from BioSpec Products (Bartlesville, OK, USA). The samples were then centrifuged at 12,000 g for 3 min, and the supernatant was transferred into clean microcentrifuge tubes for RNA and DNA extractions. The extracted RNA was resuspended in 50 µL of molecular grade water, while the DNA was resuspended in 100 μL of molecular grade water.

### 4.5. qPCR/RT-qPCR

The quantity of MC gene and transcript, *mcyA*, present in the culture was assayed via qPCR/RT-qPCR, as described in previous studies [39,47,48]. The copies·mL^−1^ was calculated using a standard curve and volume of filtered culture. The *mcyA* qPCR assay used a primer set, MSF-5′-ATCCAGCAGTTGAGCAAGC-3′ and MS2R-5′-GCCGATGTTTGGCTGTAAAT-3′ [32,33], specific to the *Microcystis mcyA* gene. The amplification reaction was done in a 20 μL reaction volume containing 10 μL of Applied Biosystem’s Power SYBR Green PCR Master Mix (ThermoFisher Scientific, Waltham, MA, USA), 2 μL of 1 mg·mL^−1^ Bovine Serum Albumin, 250 nM final concentration of forward and reverse primers, and 5 μL of sample DNA. The LOD for the qPCR assay was six gene copies·reaction⁻^1^. The reaction conditions included an initial hold of 50 °C for 2 min, followed by 95 °C for 10 min, and 40 cycles at 95 °C for 15 s and 60 °C 1 min. A standard melt curve analysis was then performed. Assays were run using Applied Biosystem’s QuantStudio^TM^ 5 Real-Time PCR System (ThermoFisher Scientific, Waltham, MA, USA). A 10-fold serial dilution of each DNA sample was performed to check for inhibition. No inhibition was detected and thus all reported quantities were from the undiluted DNA samples. A seven-point standard curve consisting of 10-fold serial dilutions in triplicate with concentrations from 5 to 5 × 10^6^ copies·mL^−1^ was performed for each run. A linearized plasmid with the *mcyA* gene fragment from *M. aeruginosa* cloned into the vector (Invitrogen™ pCR™4 TOPO^®^ TA Vector; Thermo Fisher Scientific, Waltham, MA, USA) was used for the standard. The amount of *mcyA* transcript in each RNA sample was measured using the same assay and RT-qPCR. Reverse transcription for RT-qPCR was done using Applied Biosystem’s High Capacity cDNA Reverse Transcription Kit (ThermoFisher Scientific, Waltham, MA, USA). Each reaction contained 2.5 μL of RNA, 2 μL of 10 × RT buffer, 2 μL of 10× primers, 0.8 μL of NTPs, 1 μL of Reverse Transcriptase, 1 μL of RNase Inhibitor, and 10.7 μL of molecular grade water.

### 4.6. ELISA

ELISA was used to determine the total MC-ADDA (PN 520011OH and PN520011SAES, Abraxis, Warminster, PA, USA). The assay quantifies the b-amino acid ADDA (all-*S*,all-*E*)-3-Amino-9-methoxy-2,6,8-trimethyl-10-phenyldeca-4,6-dienoic acid. Subsamples were collected in glass centrifuge tubes and frozen at −20 °C until processing and analysis. The total MC concentrations were measured by subjecting each sample to 3 freeze–thaw cycles followed by centrifugation at 3000 rpm (ThermoScientific Sorvall Legend RT^+^, ThermoFisher Scientific, Waltham, MA, USA) to pellet cellular debris. The more sensitive SAES kit was used for the growth curve experiments and had an assay range of 0.05 to 5 μg·L^−1^ of MC, and LOD of 0.016 μg·L^−1^ and LLOQ of 0.05 µg·L^−1^. For the treatment experiments, the OH version of the kit was used with an assay range of 0.15 µg·L^−1^ of MC, LOD of 0.10 µg·L^−1^, and LLOQ of 0.15 µg·L^−1^. Media blanks were measured to assess for background effects and interference. Assays were performed manually using a Biolog Microstation plate reader (Biolog, Hayward, CA, USA). Assays were performed as per the manufacturer’s protocol using the calibration curve supplied with the kit (Abraxis, Warminster, PA, USA).

### 4.7. Data Analysis

Data for comparing cell number and *mcyA* copy number for qPCR and RT-qPCR analysis were log_10_-transformed prior to statistical analysis. An *F*-test was used to compare variance between groups, and ANOVA and Student’s *t*-tests were used for pair-wise comparisons of significance. MC data from ELISA were compared using the Mann–Whitney *U*-test for significance between treatments because the MC data were not normally distributed. All of the statistical analyses, correlations, and regression analyses of the growth curves were done using Microsoft Excel for 365 Microsoft Office (16.0.14326.20936) 64-bit (Microsoft Corporation, Redmond, WA, USA). The significance level for all statistical analyses was 5%. The calculation for *Microcystis* cell growth rate (µ)·day^−1^ was made using the formula µ = ln(N/N_0_)/(t−t_0_) and mcyA copies·mL^−1^, where N ₌ number of cells at time t, N_0_ = number of cells at t_0_, t = timepoint being measured, and t_0_ = initial timepoint.

## Data Availability

The data that support the findings of this study will be public when the paper is published and are available from the corresponding author (JL) upon reasonable request as well.

## Supplementary Materials

The following supporting information can be downloaded at: https://www.mdpi.com/article/10.3390/toxins15010003/s1. Table S1: Formula for LowN BG11; Table S2: Summary of experimental conditions. Figure S1, Linear regression between mcyA gene abundance detected by qPCR (copies·mL^−1^) and microscopic counts (cells·mL^−1^). (A). Regression of the mcyA gene abundance log10 copies·mL^−1^ compared to microscopy counts log10 cell·mL^−1^ showing a correlation between gene copies and cell counts. (B) Average log10 copy·mL^−1^ and log10 cell·mL^−1^ for each sampling event; error bars represent standard deviation of duplicate samples. Figure S2, Regression of total MC concentration (µg·L^−1^) compared to (A) log10 mcyA gene abundance copies·mL^−1^ and (B) log mcyA transcript copies·mL^−1^ showing an exponential increase in MC concentration after a cell concentration of 4 log10 copy·mL^−1^ is reached. Figure S3, Effect of HAP particle size and concentration on average concentration of (A) mcyA gene abundance log10 copies·mL^−1^ and (B) MC µg·L^−1^ of Microcystis cultures treated with different sizes and concentrations of HAP. Figure S4, Effect of 40 µm HAP treatment at different concentrations on (A) mcyA gene abundance log10 copies·mL^−1^ after 21 days of growth after treatment on day seven for 2.5 g·L^−1^ or days 4, 7, 10, and 14 for 0.5 g·L^−1^ and 0.25 g·L^−1^ of HAP showing the increased HAP concentration lowered cell concentration and (B) total MC concentration measured in cultures treated with different concentrations of HAP.
