# Supplementary material for: Effective Early Treatment of Microcystis Exponential Growth and Microcystin Production with Hydrogen Peroxide and Hydroxyapatite
Source: Toxins (Basel). 2022 Dec 20;15(1):3. doi: 10.3390/toxins15010003 (PMC9864239; doi:10.3390/toxins15010003)
Supplement: Supplementary file 1 [file toxins-15-00003-s001.zip › toxins-2091328-supplementary.pdf]

toxins

MDPI

Article

Effective Early Treatment of *Microcystis* Exponential Growth and Microcystin Production with Hydrogen Peroxide and Hydroxyapatite

Ian Struewing, Nathan Sienkiewicz, Chiqian Zhang, Nicholas Dugan and Jingrang Lu

Table S1. Formula for Low N BG-11

Low N BG-11	$mg \cdot L^{-1}$
**Calcium chloride dihydrate**	36
**EDTA disodium magnesium**	1
**Magnesium sulfate • $7H_2O$**	75
**Potassium phosphate dibasic**	40
**Sodium bicarbonate**	0
**Sodium nitrate**	15
**Citric acid**	6
**Ferric EDTA**	8.6
**Zinc sulfate • $7H_2O$**	0.222
**Boric acid**	2.86
**Cobalt nitrate • $6H_2O$**	0.0494
**Cupric sulfate • $5H_2O$**	0.079
**Manganese chloride • $4H_2O$**	1.81
**Sodium molybdate • $2H_2O$**	0.39
**Sodium chloride**	1000

Table S2. Summary of experimental conditions .

Culture	Medium	Volume (mL)	Starting concentration ($cells \cdot mL^{-1}$)
**Stock**	BG-11	200	-
**Growth curves**	LowN	1000	10^2 or 10^3
**HAP particle size**	Low N	200	5×10^3
**HAP conc.**	LowN	200	10^4
**H_2O_2 conc.**	LowN	200	10^4
**H_2O_2 low cell conc.**	Low N	1000	10^2

A

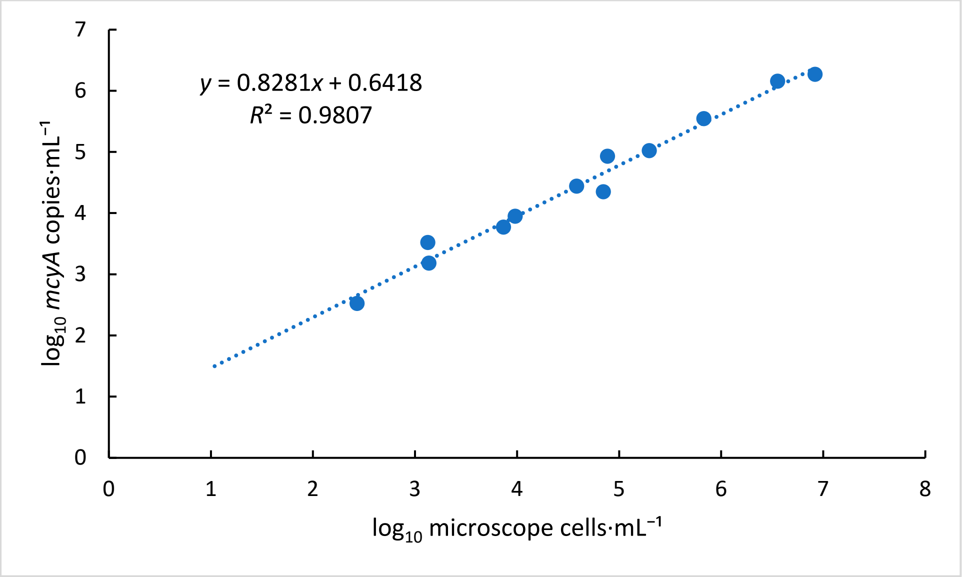

B

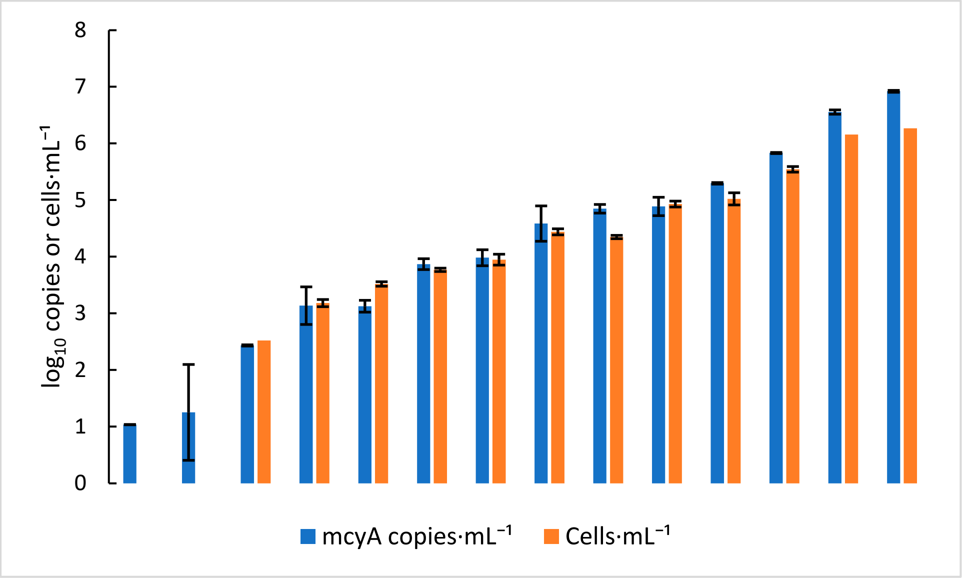

Figure S1. Linear regression between *mcyA* gene abundance detected by qPCR (copies·mL^{-1}) and microscopic counts (cells·mL^{-1}). (**A**) . Regression of the *mcyA* gene abundance $\log_{10}$ copies·mL^{-1} compared to microscopy counts $\log_{10}$ cell·mL^{-1} showing correlation between gene copies and cell counts (**B**) Average $\log_{10}$ copy·mL^{-1} and $\log_{10}$ cell·mL^{-1} for each sampling event; error bars represent standard deviation of duplicate samples.

A.

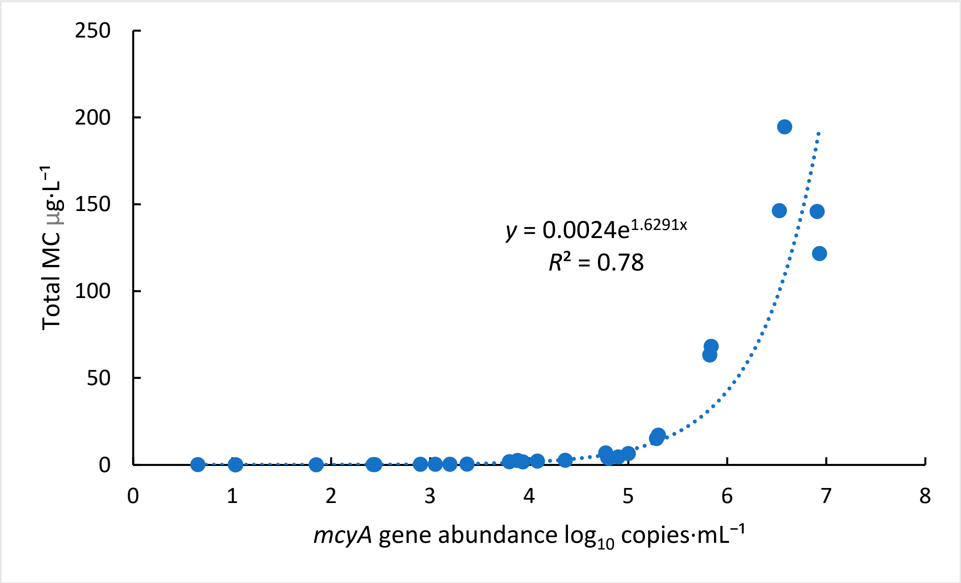

B.

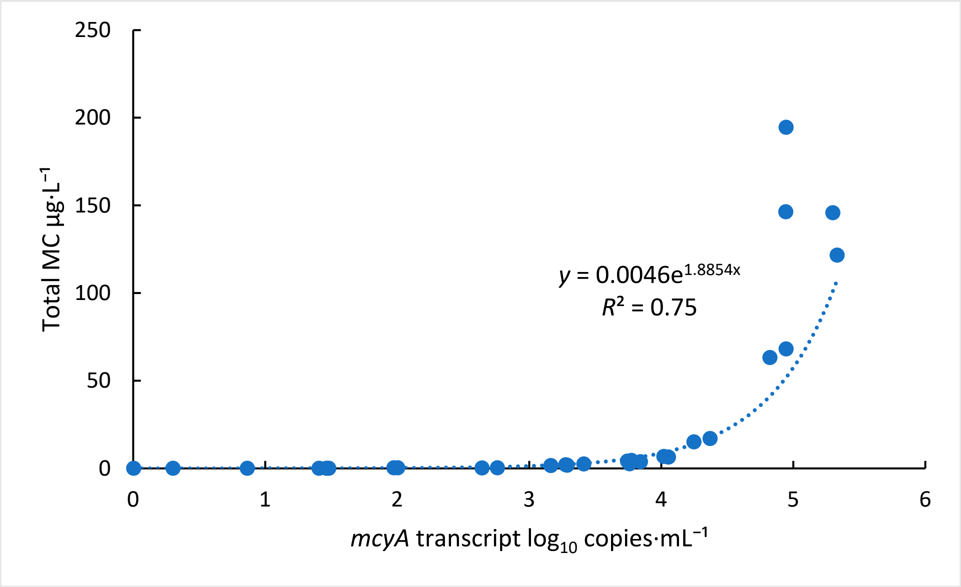

Figure S2. Regression of total MC concentration (μg·L⁻¹) compared to (**A**) $\log_{10}$ *mcyA* gene abundance copies·mL⁻¹ and (**B**) log *mcyA* transcript copies·mL⁻¹ showing exponential increase of MC concentration after a cell concentration of 4 $\log_{10}$ copy·mL⁻¹ is reached.

A.

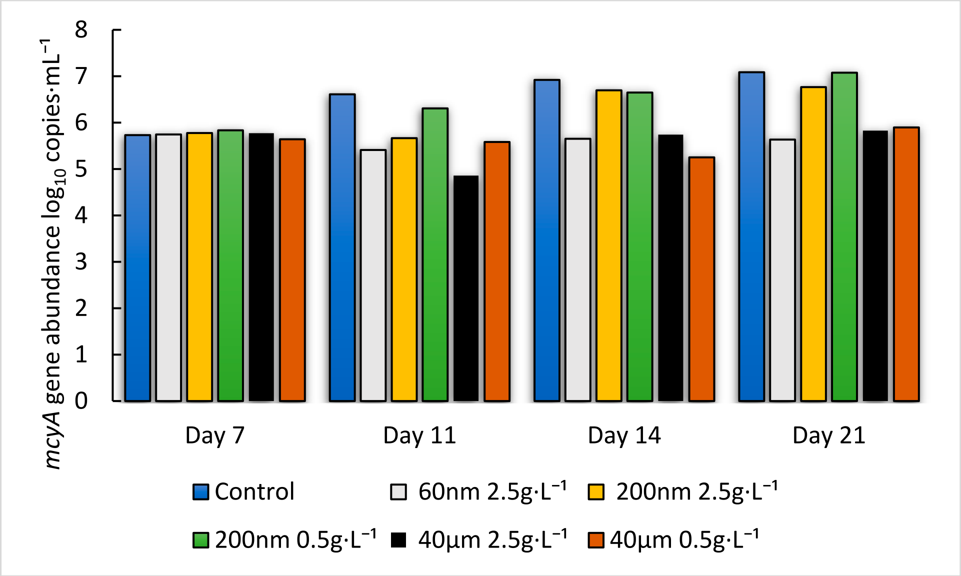

B.

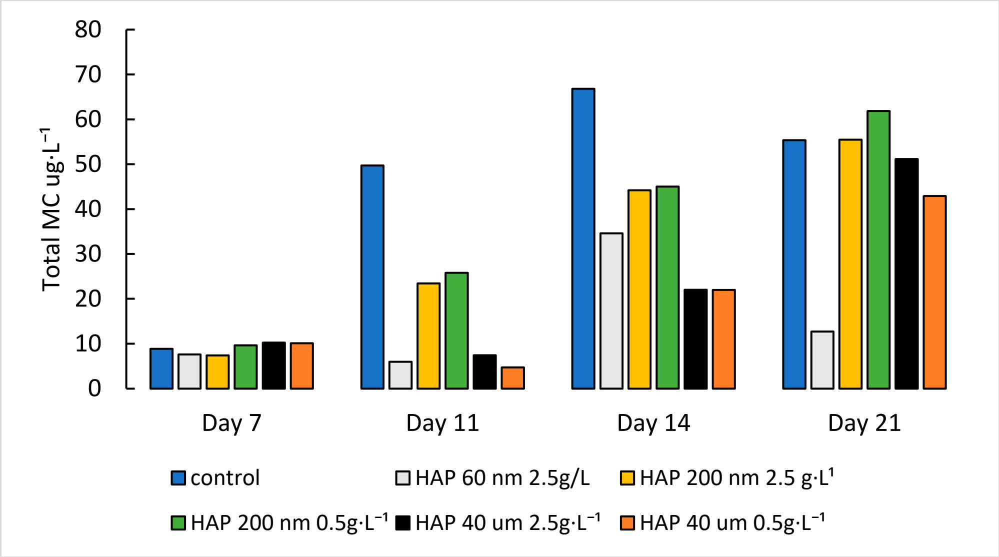

Figure S3. Effect of HAP particle size and concentration on average concentration of (**A**) *mcyA* gene abundance log_{10} copies·mL^{-1} and (**B**) MC μg·L^{-1} of *Microcystis* cultures treated with different size and concentrations of HAP.

A.

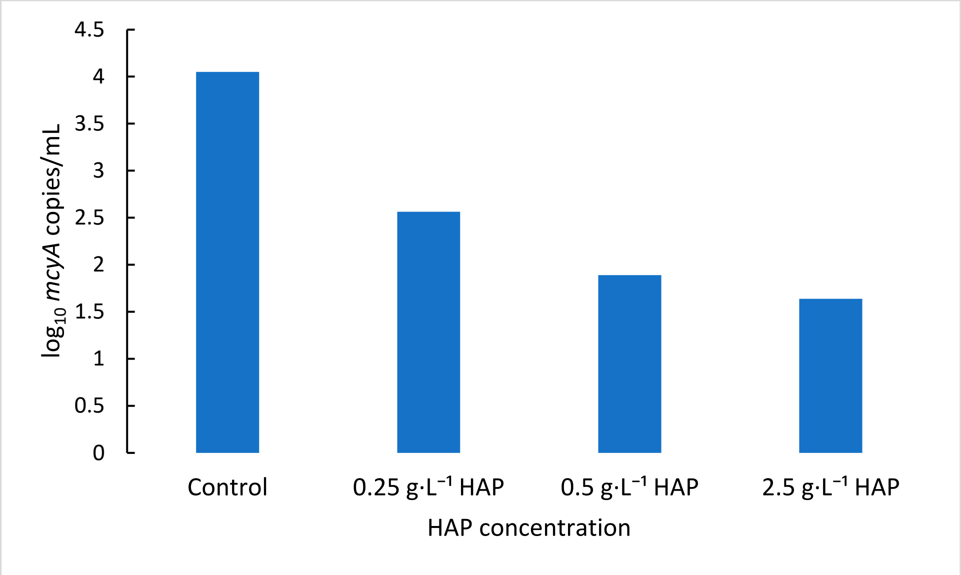

B.

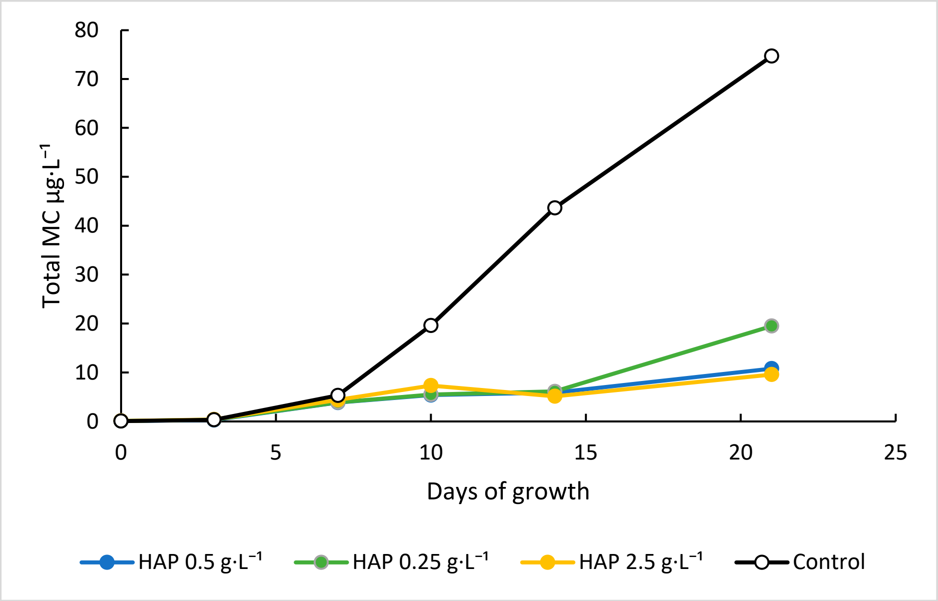

Figure S4. Effect of 40 µm HAP treatment at different concentrations on (**A**) *mcyA* gene abundance log_{10} copies·mL^{-1} after 21 days of growth after treatment on day seven for 2.5 g·L^{-1} or days 4, 7, 10, and 14 for 0.5 g·L^{-1} and 0.25 g·L^{-1} of HAP showing that increased HAP concentration lowered cell concentration and (**B**) Total MC concentration measured in cultures treated with different concentrations of HAP.

Disclaimer/Publisher's Note: The statements, opinions and data contained in all publications are solely those of the individual author(s) and contributor(s) and not of MDPI and/or the editor(s). MDPI and/or the editor(s) disclaim responsibility for any injury to people or property resulting from any ideas, methods, instructions or products referred to in the content.
